# Chlorination of (Phebox)Ir(mesityl)(OAc) by Thionyl Chloride

**DOI:** 10.3390/molecules200610122

**Published:** 2015-06-01

**Authors:** Meng Zhou, Alan S. Goldman

**Affiliations:** Department of Chemistry and Chemical Biology, Rutgers New Brunswick—Busch Campus, 610 Taylor Road, Piscataway, NJ 08854, USA

**Keywords:** chlorination, phebox ligand, iridium mesityl, thionyl chloride, pincer complex

## Abstract

Pincer (Phebox)Ir(mesityl)(OAc) (**2**) (Phebox = 3,5-dimethylphenyl-2,6-bis(oxazolinyl)) complex, formed by benzylic C-H activation of mesitylene (1,3,5-trimethylbenzene) using (Phebox)Ir(OAc)_2_OH_2_ (**1**), was treated with thionyl chloride to rapidly form 1-(chloromethyl)-3,5-dimethylbenzene in 50% yield at 23 °C. A green species was obtained at the end of reaction, which decomposed during flash column chromatography to form (Phebox)IrCl_2_OH_2_ in 87% yield.

## 1. Introduction

Carbon-chlorine bonds are widespread in pharmaceutically important organic molecules [1]. Organochlorides are also common synthetic precursors for cross-coupling [2], such as the Suzuki-Miyaura reaction [3]. In recent years C-H bond functionalization, involving initial C-H bond activation followed by functionalization of the resulting metal-carbon bond [4,5,6,7], has shown great promise in organic synthesis for constructing C-Cl bonds directly from C-H bonds [1,2,8,9,10,11,12]. These studies have typically employed a Pd catalyst and N-chlorosuccinimide, to convert aryl-H bonds to aryl-Cl bonds.

Besides Pd, Rh and Cu catalysts for carrying out arene C-H chlorination have been reported [2,13,14]. The Shilov Pt system is well-known for effecting the chlorination of methane, but studies on other substrates are rare [4,7]. An alternative method, using a Mn-porphyrin complex [15], can catalyze selective C-H chlorination without proceeding via formation of a metal-carbon bond [6].

Despite these recent advances, C-H bond chlorination is still far from being a robust and versatile synthetic method for constructing C-Cl bond [1]. The substrate scope is largely limited to aromatics and a directing group is needed to ensure regioselectivity. Development of new organometallic reactions relevant to C-H activation and C-Cl bond formation are required to overcome these challenges.

Interest in developing Ir (III) complexes for C-H bond functionalization has been growing rapidly. Crabtree and coworkers showed that Cp*Ir(chelate)X (X = monodentate anionic ligand) catalyzed the selective C-H hydroxylation of alkanes and alkyl groups by NaIO_4_ [16,17,18]. Chang and coworkers found that [Cp*IrCl_2_]_2_ catalyzed directing-group-assisted C-H amidation of arenes [19]. Nishiyama and coworkers reported the C-H activation of *n*-octane and benzene derivatives by (Phebox)Ir(OAc)_2_(OH_2_) (Phebox = 3,5-dimethylphenyl-2,6-bis(oxazolinyl)) [20]. Goldberg and co-workers subsequently found that the resulting (Phebox)Ir *n*-alkyl derivatives can undergo β-hydrogen elimination to give olefin, and that the resulting hydride can react with O_2_ to regenerate (Phebox)Ir(OAc)_2_(OH_2_) [21,22,23]. Davies and coworkers found that (Phebox)IrCl_2_(OH_2_) catalyzed asymmetric carbene C-H insertion [24].

**Scheme 1:**
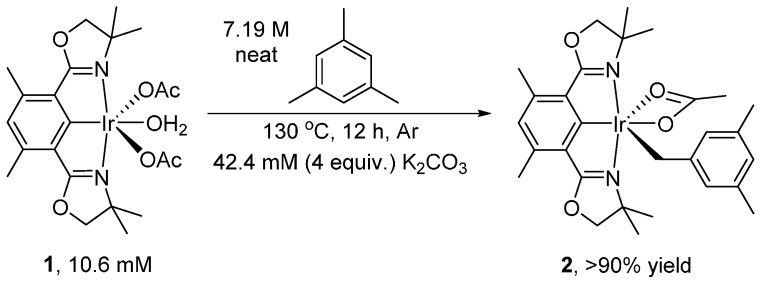
Mesitylene C-H activation by (Phebox)Ir(OAc)_2_(OH_2_) to form (Phebox)Ir(mesityl)(OAc).

We recently reported the activation and oxidation of the benzylic C-H bond of mesitylene (1,3,5-trimethylbenzene) by (Phebox)Ir complexes [25]. Exclusive benzylic C-H activation (Scheme 1) by (Phebox)Ir(OAc)_2_(OH_2_) (**1**) gave (Phebox)Ir(mesityl)(OAc) (**2**).

## 2. Results and Discussion

**Scheme 2:**
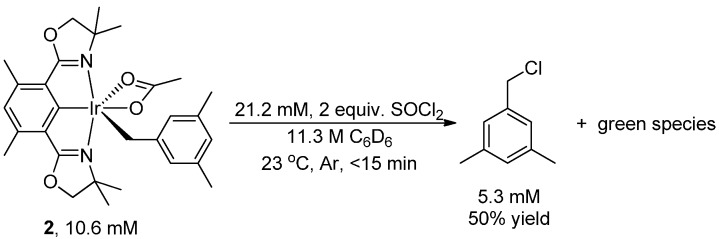
Chlorination of (Phebox)Ir(mesityl)(OAc) by thionyl chloride to form 1-(chloromethyl)-3,5-dimethylbenzene.

**Scheme 3:**
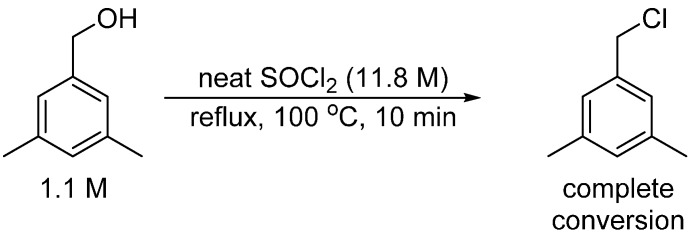
Independent synthesis of 1-(chloromethyl)-3,5-dimethylbenzene from (3,5-dimethylphenyl)methanol.

The reaction of complex **2** (10.6 mM), a red solid, with thionyl chloride (21.2 mM) resulted in the solution rapidly turning dark. The formation of 1-(chloromethyl)-3,5-dimethylbenzene in 50% yield was observed after less than 15 min at 23 °C (Scheme 2; product identity was confirmed by comparing the ^1^H- and ^13^C-NMR spectra with those of 1-(chloromethyl)-3,5-dimethylbenzene synthesized independently from purchased (3,5-dimethylphenyl)methanol, as shown in Scheme 3). C_6_D_6_ was used as solvent (Scheme 2) for direct ^1^H{^13^C} NMR analysis.

No significant (<1%) unreacted complex **2** was recovered after 15 min, indicating complete conversion of complex **2**. No significant increase (<10%) in the yield of 1-(chloromethyl)-3,5-dimethylbenzene was observed with an additional reaction time of 15 h. Using 10 equivalents of thionyl chloride (106 mM) and under otherwise identical conditions, 1-(chloromethyl)-3,5-dimethylbenzene formed in 71% yield.

Along with 1-(chloromethyl)-3,5-dimethylbenzene, a green species formed at the end of reaction (Scheme 2). We could not characterize this green species from the complex ^1^H-NMR spectrum of the reaction mixture. The green species turned purple at −196 °C when frozen by liquid nitrogen, and reverted back to green when warming back to room temperature.

**Scheme 4:**
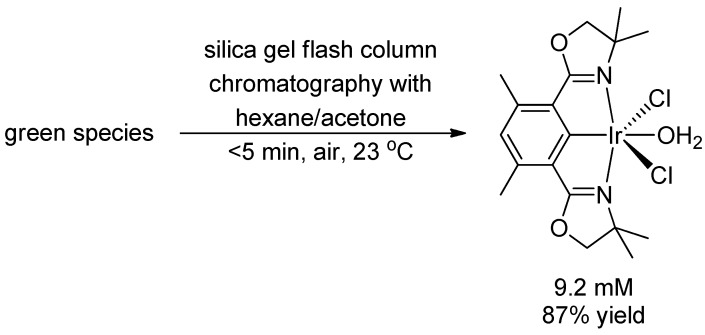
Decomposition of the green species to (Phebox)IrCl_2_(OH_2_) during silica gel flash column chromatography.

After performing silica gel flash column chromatography on the green species using acetone/hexane, we obtained a known complex [26], (Phebox)IrCl_2_(OH_2_), in 87% yield (Scheme 4). Before the chromatography no significant amount (<10%) of (Phebox)IrCl_2_(OH_2_) was present in the reaction mixture by ^1^H-NMR analysis. This suggested that the green species decomposed to form (Phebox)IrCl_2_(OH_2_) during chromatography. The result is consistent with the Phebox ligand being bound to Ir throughout the chlorination.

Relevant to this work, oxidative addition of thionyl chloride by Vaska’s complex [27,28,29,30], Ir^I^Cl(CO)(PPh_3_)_2_, gave a mixture of Ir^III^Cl_3_(CO)(PPh_3_)_2_, Ir^I^Cl(CO)(PPh_3_)_2_SO_2_ and Ir^III^Cl_2_(SOCl)(CO)(PPh_3_)_2_ ((SOCl) = chlorosulfinyl). In light of the products identified in these reports, the green species could consist of a mixture of (Phebox)Ir complexes bearing different monodentate ligands.

Currently we can only speculate on the mechanism of C-Cl bond formation, due to the complex ^1^H-NMR spectrum of the chlorination reaction mixture. The η^2^-bound carboxylate ligand in complex **2** may open to η^1^-bound to give an open coordination site for oxidative addition of thionyl chloride. A high-valent Ir complex [31,32,33,34] may form which could be responsible for C-Cl bond formation. Ison and coworkers recently reported the formation of methanol from methyl-ligated Cp*Ir^IV^(NHC) μ-oxo dimer (Cp* = C_5_Me_5_, NHC = *N*-heterocyclic carbene) by dioxygen [33,35], as an example of carbon-hetero bond formation via high-valent Ir-alkyl complex. Alternatively direct electrophilic attack on the M-C bond by thionyl chloride may occur without formation of a high oxidation state Ir complex.

## 3. Experimental Section

### 3.1. General

Solvents and reagents were purchased from VWR or Sigma Aldrich, and used without further purification. A MBraun glove box was used to store complex **2** under argon (<0.1 ppm O_2_ and <0.1 ppm H_2_O). ^1^H- and ^13^C-NMR analyses were performed on a 300, 400 or 500 MHz Varian spectrometer, using benzene solvent chemical shifts as reference at 7.16 ppm in ^1^H{^13^C} NMR spectrum (C_6_D_5_H) or 128.6 ppm for ^13^C{^1^H} NMR spectrum (C_6_D_6_). Silica gel (230–400 mesh) for flash column chromatography was purchased from SiliCycle. J-Young NMR tubes (5 mm outer diameter) were purchased from Sigma Aldrich. Reusable culture tubes (50 mL), equipped with PTFE-faced phenolic caps, were manufactured by Kimax or Pyrex. To ensure high product yield, air was rigorously removed by three cycles of freeze-pump-thaw treatment for all reactions containing Ir. The reaction yield was measured using an internal standard (1,4-bis(trifluoromethyl)benzene in C_6_D_6_) added at the end of the reaction, followed by ^1^H{^13^C} NMR analysis.

### 3.2. Chlorination of Complex **2**

Complex **2** (3.2 μmol, 10.6 mM, 2.1 mg) was dissolved in C_6_D_6_ (200 μL) in a regular 5-mm outer-diameter J-Young NMR tube in a glove box. The tube, with the J-Young valve closed, was then taken out of glove box. The valve was briefly opened under a vigorous flush of argon and an air-free (by 3 cycles of freeze-pump-thaw treatment) solution (100 μL) of thionyl chloride (63.6 mM, 6.4 μmol) in C_6_D_6_ was quickly added. The valve was quickly closed upon delivery of thionyl chloride. The red solution turned dark instantly upon addition of thionyl chloride.

After 10 min at 23 °C, the reaction mixture was analyzed directly by ^1^H-NMR spectroscopy. After obtaining the initial spectrum, an internal standard, (1,4-bis(trifluoromethyl)benzene in C_6_D_6_ was added to the reaction mixture under air for quantitation.

### 3.3. Syntheses and Characterizations


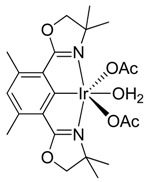


(Phebox)Ir(OCOCH_3_)_2_(OH_2_) was prepared according to literature procedures [26], starting from commercially available 1,5-bis(chloromethyl)-2,4-dimethylbenzene purchased from Sigma-Aldrich.

^1^H{^13^C} NMR chemical shifts of (Phebox)Ir(OCOCH_3_)_2_(OH_2_): 6.38 ppm (1H, arene CH, s), 3.91 ppm (4H, OCH_2_, s), 2.48 (6H, benzylic CH_3_, s), 1.8 ppm (6H, OCOCH_3_, s), 1.42 ppm (12H, aliphatic CH_3_, s).


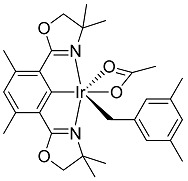


(Phebox)Ir(mesityl)(OCOCH_3_) (**2**) was made in one step from complex **1** using mesitylene under argon. Complex **1** (20 mg, 32 μmol, 106 mM), mesitylene (3 mL, 7.19 M) and K_2_CO_3_ (4 equivalents, 18 mg, 424 mM) were added to a reusable culture tube (50 mL), equipped with PTFE-faced black phenolic caps and a magnetic stir bar, in a glove box under argon. The tube was then taken out of the glove box and heated in an oil bath at 130 °C for 12 h. At the end of reaction, mesitylene solvent was removed under vacuum.

In a glove box, ether dissolved complex **2** and the solution obtained was filtered through glass wool plug packed in a glass pipette. Complex **2** was obtained in >90% yield by ^1^H{^13^C} NMR analysis in C_6_D_6_ using MeCN (64 mM) as internal standard. Product can be further purified by recrystallization in ether and pentane, at −32 °C and in a glove box.

Complex **2** in solid state appeared to be stable under argon for at least a week. Preparation in large scale (40 mg complex **2**, 64 μmol, 10.6 mM and mesitylene in 6 mL, 7.19 M) was performed in a 50 mL Reusable culture tubes (50 mL), equipped with PTFE-faced black phenolic caps. A magnetic stir bar was used.

^1^H{^13^C} NMR chemical shifts of complex **2**: 6.67 ppm (1H, ligand Ar-H, s), 6.53 ppm (1H, mesityl Ar-H, s), 6.30 ppm (2H, mesityl Ar-H, s), 3.81 ppm (2H, CH_2_O, d, *J* = 8.1 Hz), 3.65 ppm (2H, CH_2_O, d, *J* = 8.1 Hz), 2.86 ppm (2H, Ir-CH_2_-Ar, s), 2.67 ppm (6H, phebox ligand benzylic CH_3_, s), 2.16 ppm (6H, mesityl benzylic CH_3_, s), 2.04 ppm (3H, OCOCH_3_, s), 1.29 ppm (6H, two aliphatic CH_3_, s), 1.20 ppm (6H, two aliphatic CH_3_, s).

^13^C{^1^H} NMR chemical shifts of complex **2**: −1.43 ppm (s, Ir-CH_2_), 19.50 ppm (s), 21.83 ppm (s), 25.93 ppm (s), 26.44 ppm (s), 28.06 ppm (s), 66.45 ppm (s), 82.52 ppm (s), 123.6 ppm (s), 125.1 ppm (s), 127.1 ppm (s), 127.3 ppm (s), 136.4 ppm (s), 140.0 ppm (s), 150.5 ppm (s), 177.6 ppm (s), 182.4 ppm (s), 185.9 ppm (s).

X-ray crystal structure determination of complex **2** was reported previously [25].


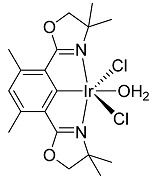


(Phebox)IrCl_2_(OH_2_) was prepared according to literature procedures [26].

^1^H{^13^C} NMR chemical shifts of (Phebox)IrCl_2_(OH_2_): 6.39 ppm (1H, arene CH, s), 3.94 ppm (4H, OCH_2_, s), 2.55 ppm (6H, benzylic CH_3_, s), 1.41 ppm (12H, aliphatic CH_3_, s).


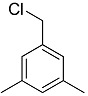


1-(chloromethyl)-3,5-dimethylbenzene was prepared from (3,5-dimethylphenyl)methanol and thionyl chloride: thionyl chloride (300 μL, 4.13 mmol, 11.8 M) and (3,5-dimethylphenyl)methanol (50 μL, 0.39 mmol, 1.1 M) were added to a NMR tube, capped. Teflon wrapping around the cap of nmr tube was used to prevent reaction mixture from leaking out. The NMR tube was heated at reflux under air (100 °C) for 10 min. After cooling to 23 °C, the nmr cap was replaced by a septum and volatiles were removed under vacuum via a needle through the septum. A solution (300 μL) of (1,4-bis(trifluoromethyl)benzene in C_6_D_6_ was added to the product mixture for ^1^H{^13^C} NMR analysis. All (3,5-dimethylphenyl)methanol was converted at the end of reaction.

^1^H{^13^C} NMR chemical shifts of 1-(chloromethyl)-3,5-dimethylbenzene: 6.75 ppm (2H, arene CH, s), 6.68 ppm (1H, arene CH, s), 4.15 ppm (2H, CH_2_Cl, s), 2.04 ppm (6H, benzylic CH_3_, s).

^13^C{^1^H} NMR chemical shifts of 1-(chloromethyl)-3,5-dimethylbenzene: 138.3 (s), 138.8 ppm (s), 130.7 ppm (s), 127.4 ppm (s), 46.97 ppm, 21.65 ppm (s).

## 4. Conclusions

Pincer Ir-mesityl complex **2**, formed by C-H activation of mesitylene, underwent facile chlorination by thionyl chloride to form 1-(chloromethyl)-3,5-dimethylbenzene. (Phebox)IrCl_2_(OH_2_) was obtained after purification of reaction mixture, indicating that the Phebox ligand remained bound to Ir throughout the course of the reaction.
